# Synergistic Effects of Icariin and Extracellular Vesicles Derived from Rabbit Synovial Membrane-Derived Mesenchymal Stem Cells on Osteochondral Repair via the Wnt/*β*-Catenin Pathway

**DOI:** 10.1155/2024/1083143

**Published:** 2024-06-22

**Authors:** Dongming Tang, Wang Tang, Huanqing Chen, Donghua Liu, Feng Jiao

**Affiliations:** ^1^ Department of Joint Surgery Guangzhou Hospital of Integrated Traditional and Western Medicine, Guangzhou, China; ^2^ Department of Spine Surgery Guangzhou Hospital of Integrated Traditional and Western Medicine, Guangzhou, China; ^3^ Guangzhou University of Chinese Medicine, Guangzhou, China

## Abstract

**Objectives:**

Osteochondral defects (OCDs) are localized areas of damaged cartilage and underlying subchondral bone that can produce pain and seriously impair joint function. Literature reports indicated that icariin (ICA) has the effect of promoting cartilage repair. However, its mechanism remains unclear. Here, we explored the effects of icariin and extracellular vesicles (EVs) from rabbit synovial-derived mesenchymal stem cells (rSMSCs) on repairing of OCDs.

**Materials and Methods:**

Rabbit primary genicular chondrocytes (rPGCs), knee skeletal muscle cells (rSMCKs), and rSMSCs, and extracellular vesicles derived from the latter two cells (rSMCK-EVs and rSMSC-EVs) were isolated and identified. The rPGCs were stimulated with ICA, rSMSC-EVs either separately or in combination. The rSMCK-EVs were used as a control. After stimulation, chondrogenic-related markers were analyzed by quantitative RT-PCR and western blotting. Cell proliferation was determined by the CCK-8 assay. The preventative effects of ICA and SMSC-EVs *in vivo* were determined by H&E and toluidine blue staining. Immunohistochemical analyses were performed to evaluate the levels of COL2A1 and *β*-catenin *in vivo*. *Results. In vitro*, the proliferation of rPGCs was markedly increased by ICA treatment in a dose-dependent manner. When compared with ICA or rSMSC-EVs treatment alone, combined treatment with ICA and SMSC-EVs produced stronger stimulative effects on cell proliferation. Moreover, combined treatment with ICA and rSMSC-EVs promoted the expression of chondrogenic-related gene, including COL2A1, SOX-9, and RUNX2, which may be via the activation of the Wnt/*β*-catenin pathway. *In vivo*, combined treatment with rSMSC-EVs and ICA promoted cartilage repair in joint bone defects. Results also showed that ICA or rSMSC-EVs both promoted the COL2A1 and *β*-catenin protein accumulation in articular cartilage, and that was further enhanced by combined treatment with rSMSC-EVs and ICA.

**Conclusion:**

Our findings highlight the promising potential of using combined treatment with ICA and rSMSC-EVs for promoting osteochondral repair.

## 1. Introduction

Osteochondral defects (OCDs) in joints are a major factor that makes joints more susceptible to damage due to a lack of neurons and blood vessels, which limits their self-renewal and healing capabilities [1, 2]. Mesenchymal stem cells (MSCs) have recently shown promise for promoting the regeneration of articular cartilage [3]. However, the use of MSC-based cartilage tissue is hampered by stringent transportation and storage conditions and also by issues of immune rejection and disease transmission [4]. Consequently, it is necessary to explore novel therapeutic treatments to overcome the shortcomings of MSC-based therapy.

Several studies have reported that the efficacy shown by MSC-based therapies was probably due to paracrine secretion derived from trophic factors instead of MSC chondrogenic differentiation [5, 6]. Extracellular vesicles (EVs), as one type of paracrine factor, are nanosized extracellular vesicles (50–150 nm in diameter) that can mediate tissue repair in pathological conditions by playing important roles in intercellular communications [7, 8, 9]. Lv et al. [10] showed that mechanical stress produced during chondrogenic differentiation causes EVs to promote the proliferation of human periodontal ligament stem cells (HPDLSCs) and their subsequent differentiation into osteoblasts. Zhang et al. [11] suggested that transplantation of EVs derived from bone marrow mesenchymal stem cells (BM-MSCs) might promote osteogenesis and angiogenesis, which play therapeutic roles in nonunion. To date, BM-MSCs have been the focus of the vast majority of cell-based cartilage repair studies [12, 13]. However, the painful collection process and low yield of MSCs obtained during each biopsy have prompted researchers to begin looking for alternative sources of MSCs [14, 15]. Recently, synovial-derived MSCs (SMSCs) have been suggested as an alternative source of MSCs. SMSCs are not only highly pluripotent but are also independent of donor age, capable of passage to the 10th generation, and can be cryopreserved [16, 17, 18, 19, 20]. SMSCs also have a superior chondrogenic capability when compared with bone marrow MSCs (BM-MSCs) [21]. Li et al. [22] showed that SMSC-based osteochondral repair facilitates the renewal of appropriate tissue texture. In addition, when compared with BM-MSCs, the proliferation of nucleated SMSCs can be increased by 60-100-fold [14, 15, 23, 24, 25], the collection process is relatively noninvasive, and fewer complications occur at the donor site [17, 26]. Lee et al. [27] reported that an SMSC-encapsulated injectable platelet-rich plasma gel showed obvious *in vivo* effectiveness for repairing damaged articular cartilage in a rabbit model. In addition, EVs isolated from KGN-pretreated rabbit suprapatellar adipose pad MSCs demonstrated a strong ability to induce the chondrogenic differentiation of stem cells and effectively promoted the proliferation of chondrocytes and expression of chondroproteins and genes [28]. Nevertheless, the effect of SMSC EVs on OCD repair remains largely unclear.

Currently, combination therapy has attracted growing attention as a method for treating OCDs [29, 30]. Icariin (ICA) is a flavonoid glycoside compound extracted from *Epimedium* and promotes against inflammation, oxidative stress, and osteoporosis [31]. Intriguingly, ICA has been identified as a potential compound for use in repairing cartilage defects [32, 33, 34, 35]. For example, ICA was found to regulate the proliferation and differentiation of chondrocytes to promote articular cartilage repair [36]. In a study of cartilage defects in rabbit knees, Zhang et al. [37] demonstrated the potential of using ICA combined with hyaluronic acid for promoting a reparative response. Subsequently, Zhang et al. [38] showed that ICA-conditioned serum combined with the biomaterial chitosan could promote the repair of OCDs in rabbit knees. In addition, whether the role of EVs in promoting osteochondral repair can be further investigated through drug intervention is also worth investigating.

Based on the above, we proposed a hypothesis that ICA combined with rSMSC-EVs may play an effective role in promoting osteochondral repair. In the presented study, we investigated the effects and mechanisms of ICA combined with rabbit SMSC (rSMSC) EVs on osteochondral repair in rabbit knee joints both *in vitro* and *in vivo*.

## 2. Materials and Methods

### 2.1. Isolation and Characteristics of rSMSCs, rPGCs, and rSMCKs

Four-week-old New Zealand rabbits (Koatech, Pyeongtaek, Republic of Korea) were housed in a room with a temperature of 20–25°C and a 12/12 hr light/dark cycle; food and water were available ad libitum. After being sacrificed with a CO_2_ euthanasia device (PVC, Wonderful Oasis Biotechnology, China), synovium tissues in the knee joint, knee cartilage tissues, and knee skeletal muscle tissues were immediately removed and cryopreserved. Next, rabbit rSMSCs [39], rabbit primary genicular chondrocytes cells (rPGCs) [40], and rabbit knee skeletal muscle cells (rSMCKs) [41] were isolated. The stemness properties of rSMSCs were verified by using flow cytometry (BD FACS Calibur) to verify the presence of specific cell surface markers (CD44, CD90, CD34, and CD45). Detection of type II collagen was used to confirm the presence of rPGCs. In brief, rPGCs were fixed with 4% polyformaldehyde and then treated with 1% Triton X-100. Next, the rPGCs were sequentially incubated with an anti-COL2A1 (1 : 500 dilution, Abcam Cambridge, MSA, USA) primary antibody and the goat antirabbit IgG H&L (Alexa Fluor® 488) secondary antibody (1 : 500, Abcam) and then photographed under a fluorescence microscope. A cell ATPase activity basic staining kit (Hengdailao Instrument Co., Ltd., Shanghai, China) was used to identify the properties of rSMCKs. All experimental protocols were approved by the Ethics Committee of Guangzhou Hospital of Integrated Traditional and Western Medicine.

### 2.2. Differentiation Induction of rSMSCs

For osteogenic differentiation induction, isolated rSMSCs were incubated in osteogenic-inducing medium (*α*-MEM + 10% FBS + 1% PS + 100 *µ*M ascorbic acid + 2 mM 2-glycerophosphate + 10 nm dexamethasone) for 14 days by using a microsphere culture system [42]; after which, they were stained with Alizarin red (cells underwent a 10 min fixation in 95% ethanol, followed by 10 min of staining with 2% alizarin red solution). For chondrogenic differentiation induction, isolated rSMSCs were used to make stem cell microspheres. After culture for 14 days, chondrogenic-inducing medium was added to the cell microspheres. After chondrogenic differentiation for 14 days, the outcome parameters were assessed by Alcian blue (Sigma, A5268) staining, as previously described [43].

### 2.3. Isolation and Identification of EVs

Based on a previous report (20), EVs from rSMSCs and rSMCKs were isolated. In brief, cells were washed and cultured in FBS-free MesenGro hMSC Medium (StemRD, San Francisco, CA, USA) for 48 hr; after which, they were centrifuged at 300x *g* for 10 min and 2,000x *g* for 10 min to remove any dead cells and cellular debris after purification by filtration, ultrafiltration (4,000x *g* for 10 min), and ultracentrifugation (100,000x *g* for 60 min), the obtained EVs (rSMSC-EVs or rSMCK-EVs) were resuspended in PBS, and used for further experiments. Finally, transmission electron microscopy (Thermo Scientific, Waltham, MA, USA) was used to identify the EVs by their morphology, and their exosomal surface markers (CD63, CD81, and TSG101) were analyzed.

### 2.4. Treatment of rPGCs

rPGCs were maintained in serum-depleted medium overnight. After reaching 80% confluence, the cells were subsequently incubated with different doses (0.1, 1, 10 *μ*g/mL) of ICA (Sigma, TX, USA) for 2 hr. Cells treated with 0.1% DMSO served as controls. To study the effects of EVs, rPGCs were classified into five groups: blank controls, rSMSC-EVs, rSMCK-EVs as negative controls, ICA, and ICA + rSMSC-EVs. To study the effects of Wnt/*β*-catenin signaling, rPGCs were divided into groups of blank controls, rSMSC-EVs (10 *µ*g/mL), ICA (10 *µ*g/mL), ICA + rSMSC-EVs, methyl vanillate (Wnt/*β*-catenin activator, 10 *μ*M), and ICA + rSMSC-EVs + KYA1797K (a Wnt/*β*-catenin inhibitor, 10 *μ*M).

### 2.5. CCK-8 Assay

Cell viability was examined using a cell counting kit-8 (CCK-8) (Dojindo, Kumamoto, Japan). In brief, rPGCs from different groups were seeded into 96-well plates (5 × 10^3^ cells/well) and cultured for 24, 48, 72, and 96 hr, respectively. Next, the cells in each well were treated with 10 *μ*L of CCK-8 solution for 2 hr at 37°C. Afterward, the optical density (OD) of each well was detected with a microplate reader (SPECTROstar Nano; BMG Labtech, Germany).

### 2.6. in Vivo Synergistic Effects of ICA and rSMSC-EVs

A total of 30 New Zealand white rabbits (female, weight range, 3–3.5 kg; age, 6 months; Chongqing Weston Biomedical Technology Co., Ltd.) were used in this study. Based on a previous study by Li et al. [22]., we constructed the OCD models in rabbit knee joints by performing lateral parapatellar arthrotomy. In the sham group (*n* = 6), a surgical incision was introduced in the right knee joint without osteochondral defect model establishment. The remaining 24 rabbits underwent surgical procedures to identify osteocartilage defects in the right knee and for further treatment. All animals were anesthetized by intravenous injection of 3% pentobarbital (30 mg/kg). For experimental purposes, the rabbits were randomly assigned to the following five experimental groups (*n* = 6 per group): (1) Sham group; (2) OCD + Saline group (rabbits had OCDs treated with Saline twice each week); (3) OCD + ICA group (rabbits had OCDs treated with ICA twice each week (0.2 mg in 0.5 mL each time)); (4) OCD + rSMSC-EVs group (rabbits had OCDs treated with rSMSC-EVs, (20 *μ*g EVs in 0.5 mL each time)); and (5) OCD + ICA + rSMSC-EVs group (OCD model rabbits were cotreated with ICA and rSMSC-EVs (0.2 mg ICA and 20 *μ*g EVs in 0.5 mL each time)). Next, six rabbits from each group were further divided into the two subgroups of three rabbits each (7-week subgroup and 14-week subgroup, respectively). In brief, the animal was placed supine on the operating table, the skin was prepared and disinfected, a midline incision was made, and a lateral parapatellar arthrotomy was performed to expose the femoral trochlea. A round full-thickness cartilage defect, 6 mm in diameter and 3 mm in depth, was created in the central portion of the femoral trochlea groove using a ring-drill (Osteochondral Autograft Transfer System; Arthrex, Inc., Naples, FL, USA). According to the experimental groups, the defects were annotated with normal saline, 0.4 mg/mL ICA solution, 0.04 mg/mL rSMSC-EVs, 0.5 mL mixed solution of 0.2 mg ICA and 20 *μ*g Evs, 0.5 mL each time, twice a week. After treatment, the patella was reduced, the joint capsule was sutured intermittently, and the wound was sutured in layers. Postoperatively, the animal is placed back in the cage and allowed to move freely in the cage without immobilization. Prior to tissue collection, each rabbit was anesthetized via intravenous administration of 3% pentobarbital (30 mg per kg) and the knee cartilage tissues were collected and evaluated for signs of disease progression. All experimental procedures involving animals were approved by the Animal Research Ethics Committee of Guangzhou Hospital of Integrated Traditional and Western Medicine and were performed in agreement with National Institutes of Health Guidelines for the Care and Use of Laboratory Animals.

### 2.7. Histological and Immunohistochemical Analyses

Samples of knee cartilage were fixed for 24 hr in neutral-buffered formalin solution supplemented with 4% formaldehyde; after which, they were subjected to 21 days of decalcification in 10% EDTA at 95°C and subsequently embedded in paraffin. The embedded tissue sections were cut into 5-*µ*m slices, deparaffinized in xylene, and then rehydrated in a gradient ethanol series. Next, the sections were washed with distilled water, stained with hematoxylin and eosin (H&E, cat: 60524ES60; Yeasen Biotechnology Co., Ltd., Shanghai, China) and toluidine blue (cat: 1046782; Haohong Pharma, Shanghai, China) and observed under a microscope. For immunohistochemical analysis, the tissue slices were incubated with anti-COL2A1 (1 : 500) and *β*-catenin (1 : 500) primary antibodies (all from Abcam, Cambridge, MA, USA), stained with diaminobenzidine peroxidase substrate, and then examined under an optical microscope (IX81; Olympus, Hamburg, Germany). In order to achieve consistent and objective results, the sections were also evaluated using the Wakitani histology scoring systems [44].

### 2.8. Quantitative RT-PCR

Total RNA was extracted from tissues using TRIzol reagent (Merck Millipore, Billerica, MA, USA) and then reverse transcribed to cDNA using a PrimeScript™ RT reagent kit with gDNA Eraser (Takara, Tokyo, Japan). Equal quantities of cDNA were subjected to PCR amplification by using Bestar® SybrGreen qPCR Master Mix (DBI) on a 7500 Realtime PCR System (Applied Biosystems, Carlsbad, CA) with the following cycling conditions: 2 min of denaturation at 95°C, followed by 40 cycles of denaturation for 20 s at 94°C, annealing for 20 s at 58°C, and extension for 20 s at 72°C. Internal reference gene U6 and Opticon-3 software were used to analyze the results. All experiments were performed in triplicate, and the primer sequences used in this study are listed in Table S1.

### 2.9. Western Blotting Analysis

Lysis RIPA buffer (Beyotime Biotechnology, China) and a BCA protein assay kit (Sigma–Aldrich, St. Louis, MO, USA) were used to extract the total proteins from tissue samples and determine the protein concentration in each extract, respectively. A 30 *µ*g sample of protein from each extract was separated by 10% sodium dodecyl sulfate-polyacrylamide gel electrophoresis (SDS-PAGE) and the protein bands were transferred onto PVDF membranes (Merck Millipore, Burlington, MA, USA), which were subsequently blocked for 2 hr with 5% nonfat milk. Next, the membranes were incubated overnight at 4°C with primary antibodies against CD63 (1 : 5,000 dilution, Abcam), CD81 (1 : 1,000, Abcam), TSG101 (1 : 4,000, Abcam), COL2A1 (1 : 1,000, Abcam), MMP-3 (1 : 10,000, Abcam), SOX-9 (1 : 5,000, Abcam), ALP (1 : 1,000, Abcam), RUNX2 (1 : 1,000, Abcam), *β*-catenin (1 : 8,000, Abcam), and GAPDH (1 : 10,000, Abcam) and then subsequently incubated with an HRP (horseradish peroxidase)-conjugated secondary antibody (1 : 10,000, Abcam) for 2 hr. Finally, the membranes were examined by using an Enhanced Chemiluminescence Kit (Merck Millipore).

### 2.10. Statistical Analysis

**Figure 1:**
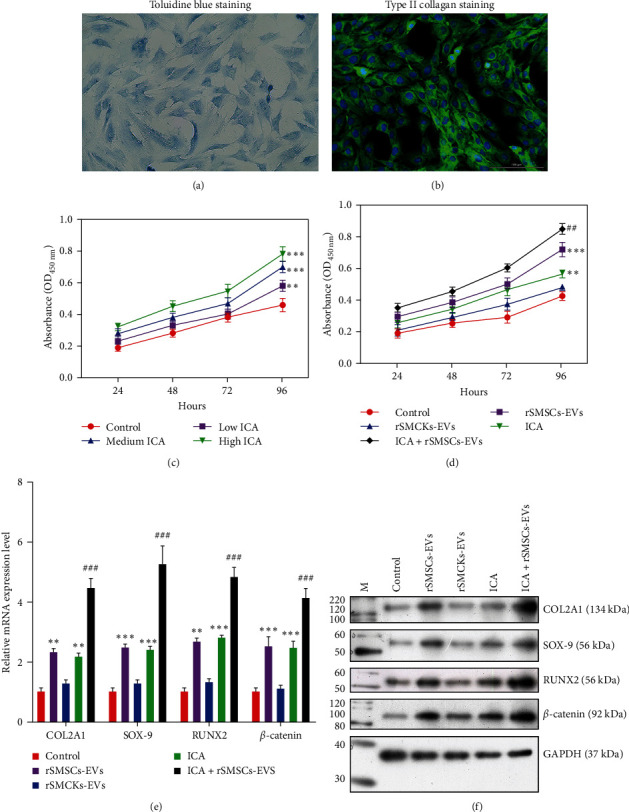
Synergistic effects of ICA and rSMSC-EVs on cell proliferation and the chondrogenic differentiation of rPGCs. (a, b) Type II collagen in rPGCs was observed by toluidine blue staining and an immunofluorescence assay. (c) CCK-8 assays were performed to determine the proliferation of rPGCs after treatment with low, medium, and high concentrations of ICA, respectively. (d) Cell proliferation was evaluated in rPGCs in the ICA, rSMSC-EVs, or combined ICA and rSMSC-exo groups. (e) Quantitative RT-PCR and (f) western blotting were used to measure the levels of COL2A1, SOX-9, RUNX2, and *β*-catenin in the ICA, rSMSC-EVs, or combined ICA and rSMSC-EVs groups.  ^*∗∗*^*p*  < 0.01,  ^*∗∗∗*^*p*  < 0.001, vs. Control; ^##^*p*  < 0.01, ^###^*p*  < 0.001, vs. ICA or rSMSC-EVs.

All statistical data were collected and analyzed by GraphPad Prism 7.0. Statistical differences for the two groups were performed with Student's *t*-test. For the comparison of three or more groups, one-way ANOVA of variance was used when there was one variable, and two-way ANOVA of variance followed by a post hoc Tukey test was used when there were two variables. Each experiment was repeated at least three times, and the results were presented as the mean ± SD. A *p*-value < 0.05 was considered statistically significant (one-way ANOVA of variance was used for Figure 1(c) and two-way ANOVA of variance was used for others).

## 3. Results

### 3.1. Differentiation of rSMSCs and Isolation of EVs

**Figure 2:**
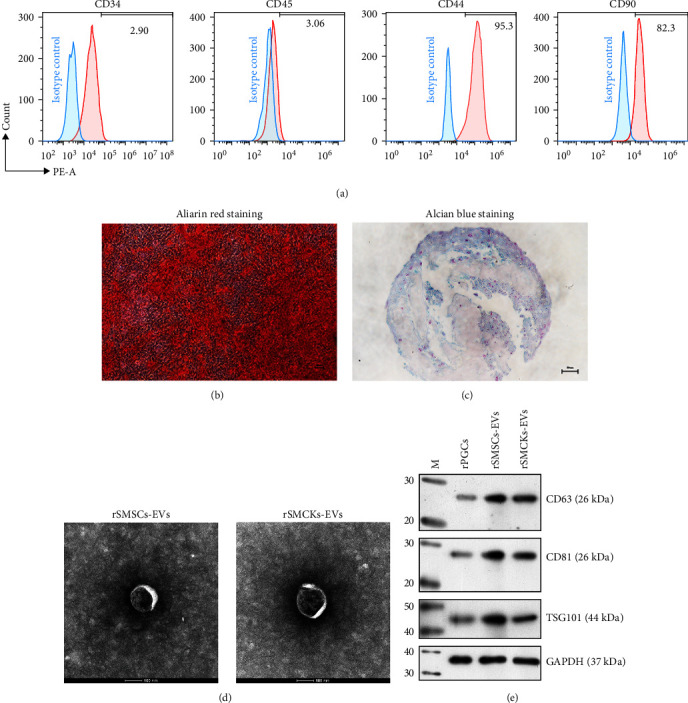
Differentiation of rSMSCs and extraction of EVs. (a) Cell surface markers in rSMSCs were identified by flow cytometry. (b) Photomicrographs of induced rSMSCs after staining with alizarin red (osteogenesis). (c) Alcian blue staining showing induced chondrogenic differentiation in rSMSC pellets on day 14 (scale bar = 50 *μ*m). (d) Observation of EVs by transmission electron microscopy. (e) CD63, CD81, and TSG101 proteins were detected by western blotting.

The rSMSCs were successfully isolated from the synovium tissues of rabbit knee joints. Flow cytometric analyses showed a >85% positive rate for CD44 and CD90, and a <5% positivity rate for CD34 and CD45 in the majority of rSMSCs (Figure 2(a)). As shown in Figure 2(b), the presence of calcium in cells was assessed based on positive staining with alizarin red. Histologically, obvious intense alizarin blue staining was exhibited in the rSMSCs pellets cultured in chondrogenic media (Figure 2(c)). Next, we isolated EVs from the conditioned media of rSMSCs and rSMCKs. As shown in Figure 2(d), transmission electron microscopy clearly revealed the presence of rSMSC-EVs and rSMCK-EVs with diameters of 40–100 nm. A western blotting analysis further verified the expression of characteristic exosomal markers (CD63, CD81, and TSG101) in rPGCs, rSMSC-EVs, and rSMCK-EVs (Figure 2(e)).

### 3.2. Synergistic Effects of ICA and rSMSC-EVs on Cell Proliferation and the Chondrogenic Differentiation Potential of rPGCs

To investigate the effects of combined ICA and rSMSC-EVs on chondrogenic differentiation, we first performed morphological observations and verified the presence of rPGCs. The cytoplasm of rPGCs exhibited intense staining with toluidine blue (Figure 1(a)). Additionally, the nuclei were stained blue and collagen type II was stained green (Figure 1(b)). Next, we determined the effective concentrations of ICA in rPGCs by using the CCK-8 assay. As shown in Figure 1(c), ICA treatment significantly increased the proliferation of rPGCs in a dose-dependent manner. After confirming the concentration of ICA, we analyzed the synergistic effects of ICA and rSMSC-EVs. We observed that treatment with either ICA or rSMSC-EVs markedly promoted the proliferation of rPGCs, and the promoting effect was further enhanced by combined treatment with ICA and rSMSC-EVs (Figure 2(d)). Notably, a significant increase in chondrogenic differentiation was observed in the combined ICA and rSMSC-EVs treatment group when compared to groups treated with ICA or rSMSC-EVs alone. Both quantitative RT-PCR and western blotting (Figures 1(e) and 1(f)) showed that the levels of COL2A1, SOX-9, RUNX2, and *β*-catenin expression were higher in both the ICA and rSMSC-EVs treatment groups when compared with the control group and were even further elevated in the ICA plus rSMSC-exo group.

### 3.3. Combined Treatment with ICA and rSMSC-EVs Promoted Cell Proliferation and Chondrogenic Differentiation via the Wnt/*β*-Catenin Signaling Pathway

**Figure 3:**
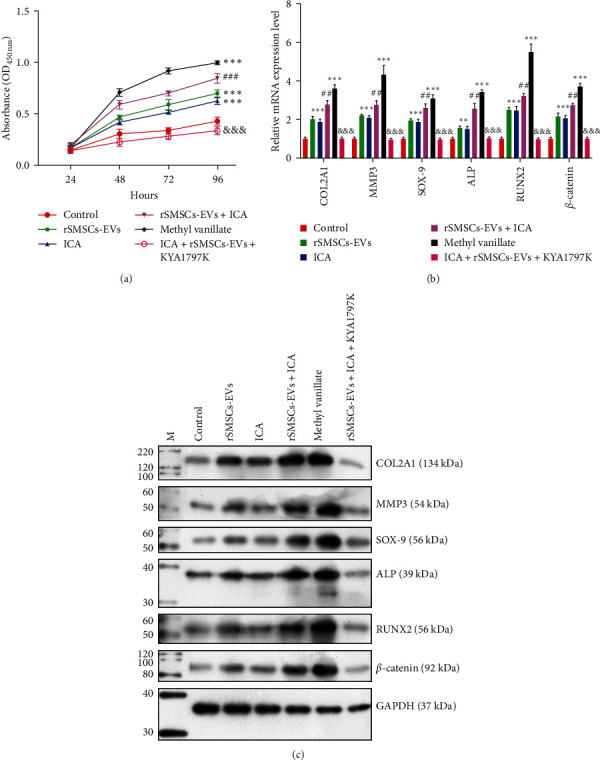
Combined treatment with ICA and rSMSC-EVs promoted cell proliferation and chondrogenic differentiation via the Wnt/*β*-catenin pathway. The rPGCs were stimulated with ICA, rSMSC-EVs, a Wnt/*β*-catenin activator (methyl vanillate), or combined with ICA + rSMSC-EVs + a Wnt/*β*-catenin inhibitor (KYA1797K), respectively. (a) CCK-8 assays were performed to detect the proliferation of rPGCs. (b) Quantitative RT-PCR and (c) western blotting were used to measure the levels of COL2A1, SOX-9, RUNX2, and *β*-catenin in rPGCs from different groups.  ^*∗∗*^*p*  < 0.01,  ^*∗∗∗*^*p*  < 0.001, vs. control; ^##^*p*  < 0.01, ^###^*p*  < 0.001, vs. ICA or rSMSC-EVs; ^&&&^*p*  < 0.001, vs. ICA + rSMSC-EVs.

The above data revealed an increase in *β*-catenin levels in the ICA and rSMSC-EVs groups, which made us speculate that the promoting effects of combined ICA and rSMSC-EVs on chondrogenesis might be correlated with the Wnt/*β*-catenin pathway. Subsequently, rPGCs were stimulated with ICA, rSMSC-EVs, a Wnt/*β*-catenin activator (methyl vanillate), or with ICA + rSMSCs-EVs + a Wnt/*β*-catenin inhibitor (KYA1797K), respectively. CCK-8 assays (Figure 3(a)) showed that treatment with ICA, rSMSC-EVs, or methyl vanillate alone could enhance the proliferation of rPGCs. A stronger effect on cell proliferation in the combined ICA and rSMSC-EVs group was notably attenuated by KYA1797K treatment. Quantitative RT-PCR (Figure 3(b)) and western blotting (Figure 3(c)) assays revealed that after methyl vanillate treatment alone, the levels of COL2A1, SOX-9, RUNX2, and *β*-catenin were significantly increased. However, KYA1797K treatment reversed the increases in COL2A1, SOX-9, RUNX2, and *β*-catenin expression induced by combined ICA and rSMSC-EVs treatment.

### 3.4. ICA and rSMSCs-EVs Combined to Repair Damaged Cartilage Tissues In Vivo

**Figure 4:**
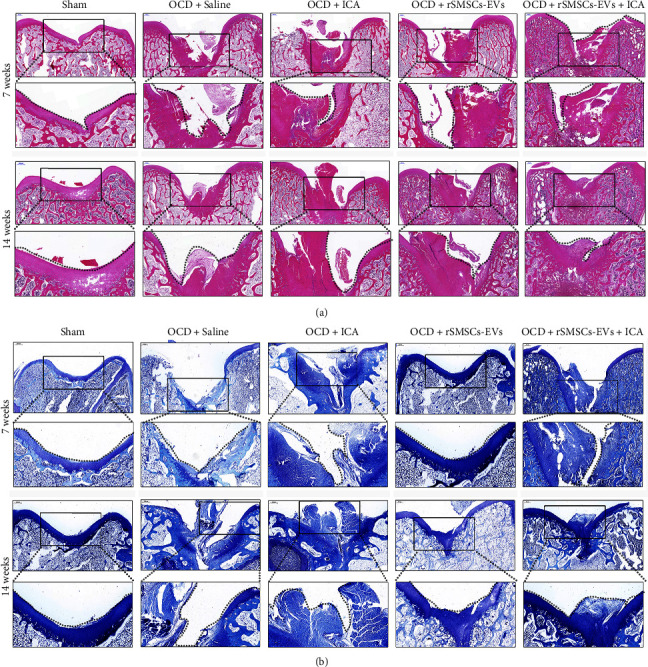
Combination treatment with ICA and rSMSC-EVs was administered to influence damaged cartilage tissues. The morphological changes in damaged cartilage tissues at 7 and 14 weeks after treatment with ICA plus rSMSC-EVs are shown by H&E staining (a) and toluidine blue staining (b).

**Figure 5:**
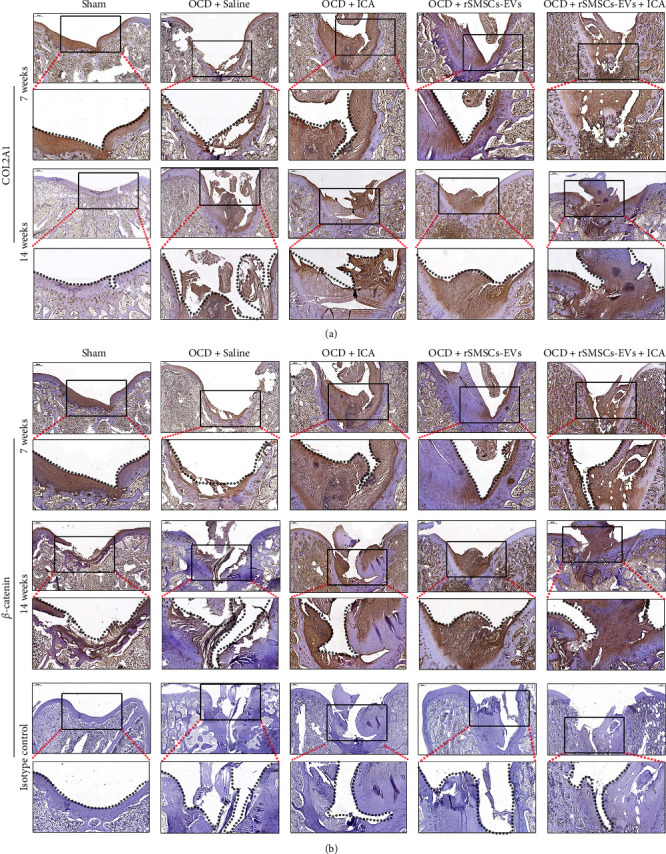
Representative images of specific proteins within the damaged cartilage tissues as detected by immunohistochemistry staining: (a) COL2A1 and (b) *β*-catenin.

**Figure 6:**
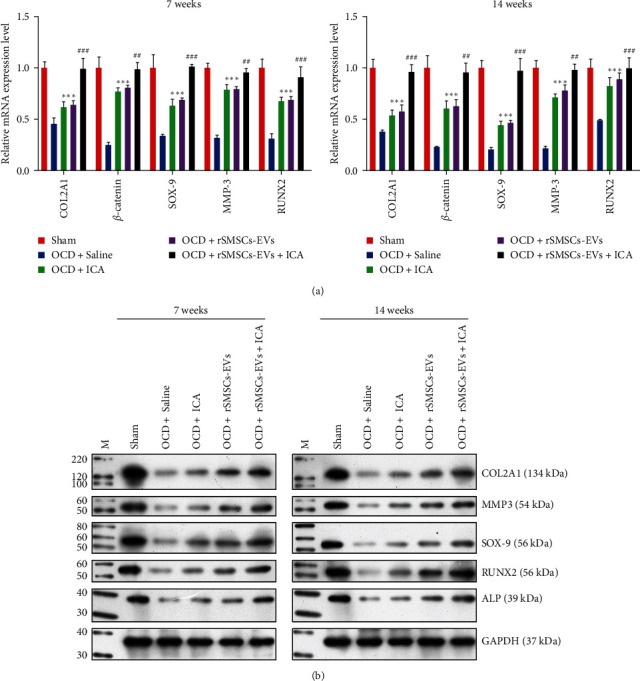
Synergistic effects of ICA and rSMSC-EVs on chondrocyte marker genes *in vivo*. Cartilage tissues were isolated from rabbits in the Sham, OCD + Saline, OCD + ICA, OCD + rSMSC-EVs, and OCD + ICA + rSMSC-EVs groups. (a) Quantitative RT-PCR and (b) western blotting were used to measure the levels of COL2A1, MMP3, SOX-9, RUNX2, and ALP expression in each group after 7 weeks and 14 weeks of treatment.  ^*∗∗∗*^*p*  < 0.001, vs. Sham; ^##^*p*  < 0.01, ^###^*p*  < 0.001, vs. OCD + ICA or OCD + rSMSC-EVs.

Next, we evaluated the repair effects of combined ICA and rSMSC-EVs on damaged cartilage tissues by constructing OCD models in rabbit knee joints. After 7 or 14 weeks of treatment with ICA, rSMSC-EVs, or their co-combination, the pathological features of damaged cartilage tissues were observed. As shown in Figure 4(a), H&E staining revealed severely damaged cartilage structure and narrowing of the cartilage layer in the model groups at 7 weeks. When compared with ICA or rSMSC-EV treatment alone, we observed narrower cracks in the cartilage injury areas, which became thicker after 4 weeks after combined treatment with ICA and rSMSC-EVs. Fourteen weeks later, we observed more obviously smooth surfaces and integrated cartilage structure in the damaged cartilage tissues treated with ICA and rSMSC-EVs. Additionally, results of toluidine blue staining showed that the contents of extracellular matrix were obviously increased after treatment with ICA plus rSMSC-EVs when compared to treatment with either ICA or rSMSC-EVs alone, and the effects were further enhanced at 14 weeks after treatment (Figure 4(b)). In addition, the Wakitani scores at 14 weeks after treatment also showed that ICA, rSMSC-EVs, ICA combined rSMSC-EVs all significantly promoted the repair of cartilage tissue, when compared with the OCD + Saline group. Moreover, ICA combined with rSMSC-EVs had the best repair effect on cartilage tissue (Table 1). Immunohistochemical analyses showed that the upregulation of COL2A1 (Figure 5(a)) and *β*-catenin (Figure 5(b)) levels in damaged cartilage tissues induced by either ICA or rSMSC-EVs treatment alone were further enhanced after treatment with combined ICA and rSMSC-EVs. At the molecular level, quantitative RT-PCR (Figure 6(a)) and western blotting (Figure 6(b)) results revealed that treatment with either ICA or rSMSC-EVs alone could significantly upregulate the expression levels of chondrogenic-related genes, including *COL2A1*, *MMP3*, *SOX-9*, *RUNX2*, and *ALP* in the damaged cartilage tissues, and combined treatment further enhanced the expression of those genes and respective protein markers after 14 weeks of treatment.

## 4. Discussion

In this study, our data showed that in the treatment of OCDs, ICA, and rSMSC-EVs had synergistic effects, which refers to the nonlinear cumulative effect of two or more active ingredients with continuous or complementary activity [45]. ICA or rSMSC-EVs alone increased the rate of cell proliferation and chondroblast differentiation, as reflected by increases of COL2A1, SOX-9, RUNX2, and *β*-catenin expression in rPGCs *in vitro*. After the treatment with combined ICA and rSMSC-EVs, the rate of cell proliferation and chondrogenic differentiation was significantly higher than that of any other group. After constructing OCD models in rabbit knee joints, we found that ICA and rSMSC-EVs worked in synergy to repair damaged cartilage tissues *in vivo*, and also to upregulate the expression of chondrogenic-related genes, including *COL2A1*, *MMP3*, *SOX-9*, *RUNX2*, and *β-catenin*. Our study demonstrated that ICA and rSMSC-EVs complement each other at the action site and enhance each other's function, and the combined treatment of the two could improve the effect of treating the damage in rabbit OCDs. Previous studies have reported that EVs derived from rabbit MSC inhibited mitochondrial dysfunction-induced apoptosis of chondrocytes [46] and promoted cartilage regeneration [47]. Obviously, the role of EVs derived from MSC and SMSC in bone tissue protection is similar.

SMSCs possess a high self-renewal capability, which is derived from the synovial membrane [16]. During synovial joint development, the synovium and articular cartilage make SMSCs more closely correlated with chondrocytes when compared with other MSCs involved joint development [48]. Because of their greater stimulatory effect on chondrogenesis, SMSCs are more suitable for cartilage repair rather than other MSCs, including BMSCs and adipose-derived MSCs (AMSCs) [49]. Previous reports have suggested a role for SMSCs in the treatment of OCDs. For example, Murata et al. [50] showed that subchondral bone in horses could be regenerated by implantation of a scaffold-free 3D-construct of SMSCs into an osteochondral defect. Lee et al. [27] suggested that SMSC-embedded platelet-rich plasma gel could successfully restore subchondral bone and resurface a defect with cartilage in a rabbit model. In line with our data, MSC-derived EVs were shown to effectively repair OCDs by promoting cell proliferation and infiltration [51]. BMSC-derived EVs promote the phenotypic transformation of synovial macrophages to relieve osteoarthritis [52]. Furthermore, we demonstrated that ICA produced an obvious effect on the proliferation and chondrogenic differentiation of rPGCs. In fact, ICA has been extensively studied in the field of regenerative medicine for its ability to inhibit osteoclastic bone resorption, facilitate matrix calcification, and increase osteogenic differentiation [53, 54, 55]. It has been suggested that bionic porous microcarriers loaded with ICA and dECM coating of bone formation derived from BMSC are both bone conductive and bone conductive and have great potential in bone repair applications [56]. At the same time, ICA regulates the proliferation and differentiation of chondrocytes to promote articular cartilage repair [36]. In rabbit knees, ICA-conditioned serum combined with hyaluronic acid showed potential for promoting a reparative response in cartilage defects [37]. Moreover, the repair effect of ICA-conditioned serum combined with biomaterial chitosan has also recently been reported [38]. When taken together, it is not difficult to conclude that combined treatment with ICA and rSMSC-EVs has superior effects in healing cartilage injuries when compared to treatment with either agent alone.

Wnt/*β*-catenin pathway is a classic pathway involved in bone metabolism regulation. When activated, it can promote the transformation of osteoblast precursor cells into osteoblasts [57]. To gain a deeper understanding of the underlying mechanism, we explored the association between the synergistic effects of ICA plus rSMSC-EVs in OCDs and the Wnt/*β*-catenin pathway. As expected, the expression levels of chondrogenic-related genes (*COL2A1*, *SOX-9*, and *RUNX2*) were significantly increased after treatment with a Wnt/*β*-catenin activator (methyl vanillate) alone. However, treatment with a Wnt/*β*-catenin inhibitor (KYA1797K) reversed the upregulation of those chondrogenic-related genes induced by combined ICA and rSMSC-EVs. These findings suggest that combined treatment with ICA and rSMSC-EVs promotes cell proliferation and chondrogenic differentiation by activating the Wnt/*β*-catenin pathway. The Wnt signaling pathway is jointly regulated by several signaling genes, and the comprehensive treatment of SMSC function and cartilage degradation through the influence of the Wnt signaling pathway. In general, the classical Wnt/*β*-catenin is inhibited in the osteoarthritis synovium, while the nonclassical PCP and Wnt/Ca^2+^ pathways are activated. Huang et al. [58] indicated that WNT5A significantly aggravated joint degradation, while WNT10A had an obvious antiaging effect on SMSCs. The activation of the Wnt/*β*-catenin signaling pathway can inhibit the lipogenesis of BM-MSCs and promote osteogenic differentiation [59], knock out the SMSC in *β*-catenin will lead to the decline in bone mass and bone microstructure, associated with delayed fracture healing [60]. It has also been reported that SMSC-EVs can reduce chondrocyte damage during osteoarthritis through miR-130b-3p mediated LRP12/AKT/*β*-catenin axis inhibition. SMSC-derived exosome microRNA-320c promotes cartilage damage repair in osteoarthritis rats by targeting ADAM19-dependent Wnt signaling pathways [61]. As a crucial regulator of skeletal development and remodeling, Wnt/*β*-catenin signaling might mediate the effects of Indian Hedgehog (IHH) signaling, which is highly activated in cartilage during osteoblast differentiation [62]. Moreover, a recent report by Oichi et al. [63] indicated that *β*-catenin-dependent Wnt signaling plays an important role in mediating the differentiation of skeletal mesenchymal cells toward a chondrogenic or osteogenic direction. Consistent with our data, Ma et al. [64] showed that EVs derived from adipose-derived stem cells (ADSC-EV) activated Wnt/*β*-catenin signaling to promote cutaneous wound healing. ICA activates the Wnt/*β*-catenin signaling pathway by regulating miR-23a, which promotes BSMC osteogenesis and adipogenesis [65]. In addition, ICA can activate Wnt/*β*-catenin signaling to promote the differentiation of BMSCs into chondroblasts [66]. The strategy of targeting the Wnt/*β*-catenin signaling pathway to improve osteoarthritis deserves further investigation.

In summary, our study demonstrated that cotreatment with ICA and rSMSC-EVs greatly improved rabbit OCD therapy outcomes by activating the Wnt/*β*-catenin pathway. After considering the ability of ICA to promote cell proliferation and the expression of chondrogenic-related genes, we conclude that ICA exerted supplementary beneficial effects on MSC-based therapies in our rabbit model of OCDs. Moreover, without the risk of immune rejection and disease transmission, and the limitation of transportation and storage, rSMSC-EVs have more therapeutic application prospects compared with the MSC-based cartilage tissue. Therefore, the combined use of ICA and rSMSC-EVs is a potential strategy for promoting osteochondral repair and may have applications in tissue engineering. Furthermore, we believe that rSMSC-EVs loaded with ICA in treatment of OCDs deserve in-depth research in the future. However, failure to consider the impact of ICA on EVs of SMSC and other cells in rabbits may be one of the limitations of this study. The use of EVs fluorescence labeling technology in animal experiments to detect the uptake of EVs by chondrocytes may make the experimental results more convincing.

## Figures and Tables

**Table 1:** Histologic evaluation by Wakitani histology scoring systems in the 14th week.

Parameter (Wakitani)	Sham	OCD + Saline	OCD + ICA	OCD + rSMSCs-EVs	OCD + rSMSCs-EVs+ICA
Cell morphology	0.07 ± 0.10	3.33 ± 0.47	2.6 ± 0.14	2.2 ± 0.28	1.07 ± 0.09
Matrix staining	0.10 ± 0.08	1.77 ± 0.56	1.67 ± 0.47	1.33 ± 024	0.98 ± 0.02
Surface regularity	0.33 ± 0.24	2.81 ± 0.14	2.07 ± 0.17	1.33 ± 0.24	1.50 ± 0.03
Thickness of the cartilage	0.04 + 0.06	1.96 ± 0.04	0.68 ± 0.45	1.07 ± 0.09	0.45 ± 0.07
Integration of donor with host	0 ± 0	1.93 ± 0.09	1.44 ± 0.08	1.17 ± 0.12	0.57 ± 0.09
Total	0.54 ± 0.19	11.82 ± 0.94	8.46 ± 0.83	7.10 ± 0.40	4.60 ± 0.28

*Abbreviations*. OCD, Osteochondral defects; ICA, icariin; rSMSCs, rabbit synovial membrane-derived mesenchymal stem cells; EVs, extracellular vesicles; and SD, standard deviation. Statistical analysis is not provided because the grading system is not linear or comparably equivalent between the five categories.

## Data Availability

The datasets used to support the findings of this study are available from the first author upon request.

## Supplementary Materials

Supplementary Materials Table S1: primers for quantitative real-time PCR.
