# Supplementary material for: Synergistic Effects of Icariin and Extracellular Vesicles Derived from Rabbit Synovial Membrane-Derived Mesenchymal Stem Cells on Osteochondral Repair via the Wnt/β-Catenin Pathway
Source: Anal Cell Pathol (Amst). 2024 Jun 22;2024:1083143. doi: 10.1155/2024/1083143 (PMC11214593; doi:10.1155/2024/1083143)
Supplement: Supplementary Materials — Table S1: primers for quantitative real-time PCR. [file 1083143.f1.docx]

**Table S1.** Primers for quantitative real-time PCR.

| **Gene** | **Forward (5’ - 3’)** | **Reverse (5’ - 3’)** |
| --- | --- | --- |
| COL2A1 | GGTCTGGGATGGAAACTGTG | TCCTTTCTGCCCCTTTGGTC |
| MMP-3 | TCAAGGGATGCAGACACCAC | TCACCTCCAAGCCAAGGAAC |
| SOX-9 | AAGATGACCGACGAGCAG | GGCTTGTTCTTGCTGGAG |
| ALP | ACTTTGTCTGGAACCGCACT | GTGGTCAATCCTGCCTCCT |
| RUNX2 | GATGACGTCCCCGTCCATTC | GGAACAGGGTGGTGGAAGAC |
| β-catenin | ATGACTCGAGCTCAGAGGGT | GATTGCACGTGTGGCAAGTT |
| GAPDH | CACCCACTCCTCTACCTTCG | GGTCTGGGATGGAAACTGTG |
